# High prevalence of asthma symptoms in Warao Amerindian children in Venezuela is significantly associated with open-fire cooking: a cross-sectional observational study

**DOI:** 10.1186/1465-9921-14-76

**Published:** 2013-07-20

**Authors:** Stèphan Kraai, Lilly M Verhagen, Enrique Valladares, Joaquin Goecke, Lorena Rasquin, Paula Colmenares, Berenice Del Nogal, Peter WM Hermans, Jacobus H de Waard

**Affiliations:** 1 Laboratory of Pediatric Infectious Diseases, Radboud University Medical Centre, PO Box 9101 (internal post 224), 6500 Nijmegen, HB, The Netherlands; 2 Laboratorio de Tuberculosis, Instituto de Biomedicina, Caracas, Venezuela; 3 Facultad de Medicina, Universidad Central de Venezuela, Caracas, Venezuela; 4 Departamento de Pediatría, Hospital de Niños J.M. de los Ríos, Caracas, Venezuela

**Keywords:** Asthma[Mesh], Wheeze, Woodsmoke, Tobacco smoke pollution[Mesh], Smoke[Mesh], Indigenous children

## Abstract

**Background:**

The International Study on Asthma and Allergies in Childhood (ISAAC) reported a prevalence of asthma symptoms in 17 centers in nine Latin American countries that was similar to prevalence rates reported in non-tropical countries. It has been proposed that the continuous exposure to infectious diseases in rural populations residing in tropical areas leads to a relatively low prevalence of asthma symptoms. As almost a quarter of Latin American people live in rural tropical areas, the encountered high prevalence of asthma symptoms is remarkable. Wood smoke exposure and environmental tobacco smoke have been identified as possible risk factors for having asthma symptoms.

**Methods:**

We performed a cross-sectional observational study from June 1, 2012 to September 30, 2012 in which we interviewed parents and guardians of Warao Amerindian children from Venezuela. Asthma symptoms were defined according to the ISAAC definition as self-reported wheezing in the last 12 months. The associations between wood smoke exposure and environmental tobacco smoke and the prevalence of asthma symptoms were calculated by means of univariate and multivariable logistic regression analyses.

**Results:**

We included 630 children between two and ten years of age. Asthma symptoms were recorded in 164 of these children (26%). The prevalence of asthma symptoms was associated with the cooking method. Children exposed to the smoke produced by cooking on open wood fires were at higher risk of having asthma symptoms compared to children exposed to cooking with gas (AOR 2.12, 95% CI 1.18 - 3.84). Four percent of the children lived in a household where more than ten cigarettes were smoked per day and they had a higher risk of having asthma symptoms compared to children who were not exposed to cigarette smoke (AOR 2.69, 95% CI 1.11 - 6.48).

**Conclusion:**

Our findings suggest that children living in rural settings in a household where wood is used for cooking or where more than ten cigarettes are smoked daily have a higher risk of having asthma symptoms.

## Background

Health authorities have largely ignored asthma as a cause of respiratory distress in children from Latin America. Seventeen centers in nine different Latin American countries participated in the International Study of Asthma and Allergies in Childhood (ISAAC) Phase One [1,2], aimed at reporting the prevalence of respiratory symptoms related to asthma. Prevalence rates of asthma symptoms of 8.6 to 32.1 percent were observed in the 52,549 written questionnaires in children aged 13 to 14 years and the 36,264 written questionnaires in 6 to 7 year-olds. One of the conclusions was that asthma symptoms could no longer be considered a minor health problem in Latin America, because the prevalence of asthma symptoms in Latin America was as high and variable as prevalence rates observed in other regions of the world [3]. A more recently performed ISAAC study including over one million children from countries all over the world concluded that the prevalence of asthma symptoms was highest in English language countries and Latin America [4].

Asthma is one of the most important diseases of childhood, causing substantial morbidity. A number of studies performed in industrialized countries in the 1990s showed prevalence rates of asthma symptoms increasing 1.41 - 1.56 fold compared with the same cohorts in the 1960s and 1970s [5-8]. There are different hypotheses about the etiology of asthma. In 1989, Strachan was the first scientist to propose a relationship between asthma and exposure to infectious agents. He observed that the allergic disease hay fever was less common in children from larger families compared with families with only one child. He suggested that exposure to infectious agents protects against the development of hay fever [9]. Strachan's "hygiene hypothesis" suggests that microbial exposure during a critical time window in utero or early infancy leads to selective T-cell differentiation and protection against later atopy and asthma. In rural populations, factors such as gastrointestinal bacterial infections, parasitic infections, poor hygiene, a high number of children per family and a high burden of severe acute viral infections in infancy are common [3]. According to the hygiene hypothesis, these factors have a protective effect against asthma in children living in rural settings. However, high prevalence rates of asthma symptoms in children from poor Latin American families were observed in the ISAAC study [3], suggesting that other factors play a role in the development of asthma symptoms.

Approximately half the world’s population and up to 90% of rural households in developing countries still rely on unprocessed biomass fuels in the form of wood, dung and crop residues. These are typically burned indoors in open fires or poorly functioning stoves, often causing extreme pollution [10]. Young children are often carried on their mothers’ backs during cooking and therefore spend many hours inhaling smoke. Despite the large population exposed, and the fact that asthma is now the most common chronic disease among children worldwide, only a handful of studies have explored the potential association between asthma symptoms and indoor air pollution in rural settings. Several reports from rural areas observed a significantly higher risk of asthma symptoms in children from households where open biomass fire is used for cooking compared with households with other types of stoves with adjusted odds ratio (AOR) varying from 2.2 to 4.9 [11-13]. However, other studies have reported a lack of a significant association between asthma symptoms and biomass smoke [14-17]. In support, a large ISAAC study including 2,430 South African children did not report a significant association of asthma symptoms with cooking fuel type [18]. Von Mutius even found evidence of a protective effect of biomass smoke on atopy (AOR 0.67, 95% confidence interval (CI) 0.49 - 0.93) and bronchial hyper-responsiveness (AOR 0.55, 95% CI 0.34 - 0.90) in children living in homes where wood was used for heating in rural Germany [19]. A possible reason for these contradictory results is that most studies investigating associations between asthma symptoms and indoor air pollution are small observational studies, often with methodological difficulties [20].

It is well confirmed that exposure to passive tobacco smoke in childhood increases the risk of asthma symptoms and allergic disease. Gold et al. stated in their literature review that environmental tobacco smoking, and particularly maternal smoking, increases the risk of asthma symptoms in children less than 6 years of age [21]. The effect of exposure to tobacco smoke on the prevalence of asthma symptoms in children has been studied extensively, however, most of these studies were performed in urban settings [21-24]. Noonan et al. included 396 children from grade one till eight from two rural villages in the United States. They found an AOR of 2.39 (95% CI 1.35 - 4.24) for wheezing in the last 12 months among children living in households with reported tobacco usage compared to children living in households where no tobacco was used. Furthermore, they reported a stronger effect of tobacco smoke in grade school children compared to middle school children [25]. The lack of knowledge on risk factors for asthma symptoms in non-developed countries is striking considering the large body of research that has been performed in developed countries. We performed a cross-sectional survey to study the effects of indoor wood smoke and indoor tobacco smoke on the prevalence of asthma symptoms in Warao Amerindian children.

## Methods

### Study setting

The Warao people are the second most important Native American group in Venezuela. They inhabit the Delta Amacuro located close to the Atlantic Ocean on the eastern coast of Venezuela. The Warao live in about 300 geographically isolated villages that are spread throughout this area, where they receive little medical attention and live under precarious sanitary conditions, experiencing a high incidence of infectious diseases [26]. The Warao originally cooked on open wood fires (*leña)*. The women gather wood and lumber for cooking on open fireplaces. Nowadays, there are parts of the Delta Amacuro where gas stoves are common. However, there is a limited supply of gas in this rural watery area. Therefore it is common to use both gas as well as wood burning for cooking. The Warao people usually live together with the whole family in houses raised on stilts called “Janoko”. In general, mothers do not smoke, while fathers and grandparents often smoke. Both cigarettes as well as homemade tobacco pipes are used.

### Ethical considerations

Oral informed consent was obtained from all parents or guardians after explaining the nature and objectives of the study in Spanish and/or in their native language. The ethical committee of the Instituto de Biomedicina, the Regional Health Services, and the Delta Amacuro Indigenous Health Office (Servicio de Atención y Orientación al Indígena) approved this survey.

### Data collection

**Figure 1:**
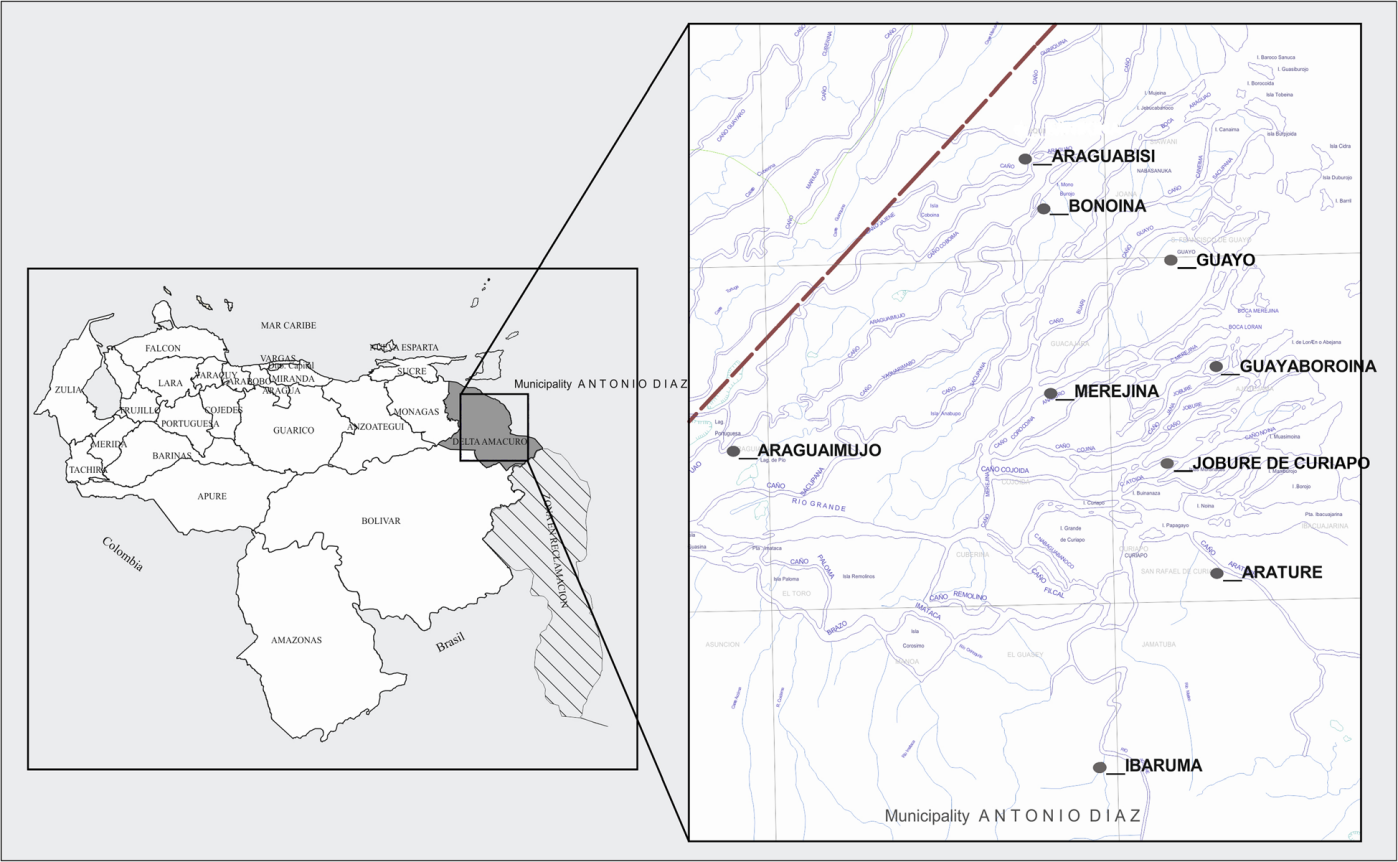
Map of Delta Amacuro.

This cross-sectional survey was carried out during the rainy season from June 1, 2012 to September 30, 2012, in nine isolated Warao villages in the municipality of Antionio Diaz (Figure 1). In the analysis the nine villages were grouped in parishes. The Curiapo parish contains Arature, Jobure the Curiapo and Ibaruma. Manuel Renaud contains Araguabisi and Bonoina. Padre Barral contains Merejina, Guayo and Guayaboroina. Santos de Abelgas contains Araguaimujo. Initially, all children attending health clinics were included. Subsequently, door-to-door visits were conducted in each of the nine communities. The investigators stayed in every community for approximately three to seven days. If an individual was not home during the door-to-door visits, return visits were made to include all age-eligible children (2–10 years) living in the communities at the time of the survey. Information was collected by a structured face-to-face interview with parents or guardians of the children based on the standardized validated ISAAC questionnaire [2]. All eight questions from its core questionnaire for wheezing and asthma were translated into Spanish and included in the questionnaire. In addition to these items, information was collected on the age and sex of the child, the method of cooking used in the household, whether the house had walls and the number of smokers living in the household as well as the number of cigarettes smoked daily by each smoker. If parents or guardians did not speak Spanish, the questions were translated into the native Warao language by local translators. Prior to the start of the survey, the study aims and the definitions and explanations of the terms used were discussed with community elders, community health workers and local translators. Preliminary testing of the questionnaires was then carried out by the local translators together with the survey staff. This indicated that some questions required clarification; therefore, Spanish words were added to the questionnaire. Specifically, the word "wheezing" was explained as a whistling sound or a high pitched sibilant sound heard in the neck but coming from the chest and the word “respiratory infection” was explained as “a cold or pneumonia” because “respiratory infection” is often interpreted locally as a production of lung sounds, including wheezing or sibilant rhonchi. Furthermore, “dry cough” was explained as “a cough without coughing up phlegm” and “attack of wheezing” was explained as an episode or occasion of wheezing as the term “attack” was associated locally with a sudden rather than gradual onset of symptoms. If parents or guardians did not understand terms such as “whistling sound in the chest”, as discussed in the meetings held prior to the initiation of the survey, the local translators explained the terms to them in their local Warao language*.* The date of birth was taken from a written record when available, usually a birth certificate or a vaccination card. If birth dates were not recorded or known with certainty, the caregiver was asked to give an approximate date of birth. Data collection was performed by a physician together with one Dutch and four Venezuelan medical students. Epidemiological studies have used different methods of measuring asthma prevalence and its symptoms in surveys and these differences can have a large impact on estimates of prevalence [27]. In 1991 the ISAAC was set up to achieve uniform diagnostic criteria. We defined asthma symptoms as self-reported wheezing in the last 12 months, in concordance with the ISAAC criteria [2].

### Statistical analysis

Categorical variables were analyzed using the Chi-square test or Fisher’s exact test, as appropriate. To assess the relationship between categorical variables and the prevalence of wheezing in the last 12 months, ORs and 95% CIs were calculated by means of univariate and multivariable logistic regression analyses. Only variables with a p-value ≤ 0.25 in the univariate analysis were entered into a multivariable regression model. We included the following variables in the multivariable regression model: age, sex, region, cooking method, wall status of the house and number of cigarettes per day smoked in the household.

The SPSS program for Mac version 20.0.0 (SPSS Inc, Chicago, IL) was used for statistical analyses.

## Results

All parents or guardians were cooperative and willing to answer questions. From June 1, 2012 to September 30, 2012, 630 children two to ten years of age were included. The median age of included children was 6 years (interquartile range 4–8). Asthma symptoms were recorded in 164 of these children, giving a prevalence of 26 percent.

In our study population, 48 percent of the children lived in households where open wood fires were used for cooking. Thirty-one percent of the included children lived in a household where a combination of gas and wood was used for cooking and 20 percent in a household where only gas was used for cooking. Gasoline was used for cooking in one household of three children. The prevalence of wheezing in the last 12 months differed by cooking method while the prevalence of wheezing at any point in time, exercise-induced wheezing or nocturnal cough did not differ by cooking method (Table 1). Seventy-six percent of the children living in a house without walls were exposed to cooking with wood compared to only 36 percent of the children living in a house with walls (p < 0.01).

**Table 1:** Prevalences of asthma symptoms by cooking method

**Written questionnaire**	**Total (n, %)**	**Gas (n, %)**	**Gas and wood (n, %)**	**Wood (n, %)**	**p-value**
Wheezing “ever” (n = 631*)	266 (42)	46 (37)	78 (38)	142 (47)	0.09
Wheezing in last 12 months (n = 627)	164 (26)	19 (15) ^a^**	40 (20) ^ab^	105 (35) ^b^	**<0.01**
Diagnosed Asthma (n = 591)	108 (18)	20 (17)	34 (18)	54 (19)	0.87
Exercise-induced wheeze last year (n = 621)	89 (14)	9 (7)^ a^	17 (9)^ a^	63 (21)^ b^	**<0.01**
Nocturnal cough last year (n = 608)	163 (27)	29 (24)	50 (25)	84 (29)	0.51
Number of wheezing episodes last year (n = 626)					**<0.01**
0	463 (74)	104 (85) ^a^	163 (80) ^a^	196 (65) ^b^	
1-3	119 (19)	16 (13) ^a^	37 (18) ^a^	66 (22) ^a^	
4-12	34 (5)	3 (2) ^ab^	2 (1) ^b^	29 (10) ^a^	
More than 12	9 (1)	0 (0) ^a^	1 (0) ^a^	8 (3) ^a^	
Sleep disturbance due to wheezing last year (n = 620)					0.07
Never	535 (86)	114 (94)	176 (88)	245 (82)	
Less than once a week	77 (12)	6 (5)	23 (12)	48 (16)	
Once or more than once a week	8 (1)	1 (1)	1 (1)	6 (2)	

* n represents the number of cases in which an answer other than “unknown” was recorded on the questionnaire.

**Pairwise post-hoc comparisons: the same letters in the same row indicate absence of significant statistical difference by the Chi-square test or Fisher’s exact test, as appropriate. For example the prevalence of “Wheeze in last 12 months” in the “Gas” group (a) was not statistically significantly different from the prevalence in the “Gas and Wood” group (ab) while it was significantly lower than the prevalence observed in the children in the “Wood” group (b).

Significant p-values are indicated in bold.

Of the included children, 49 percent were exposed to passive tobacco smoking with the number of cigarettes smoked in the household daily varying from one to 44. Sixty-seven percent of the children had a smoking father and nine percent of the children had a smoking mother. The mother was the primary caregiver in 92 percent of the cases and the father was the primary caregiver in one percent. The relationship of the smoker to the child was not significantly associated with the prevalence of asthma symptoms (Table 2).

**Table 2:** Multivariable analysis of factors associated with wheezing in the last 12 months

			**Univariate analysis**		**Multivariable analysis**
**Characteristic**	**n (%)**	**% wheezing last 12 months**	**OR (95% CI)**	**p-value**	**AOR (95% CI)**
Age (years)				<0.01	
2 - 3	154 (25)	36	1		1
4 - 5	157 (25)	24	0.54 (0.33 - 0.88)		0.51 (0.30 - 0.86)
6 - 7	150 (24)	26	0.62 (0.38 - 1.01)		0.68 (0.40 - 1.14)
8 - 10	167 (26)	19	0.42 (0.25 - 0.70)		0.46 (0.27 - 0.78)
Sex				0.037	
Male	316 (50)	30	1		1
Female	312 (50)	22	0.68 (0.48 - 0.98)		0.74 (0.50 - 1.08)
Region				<0.01	
Curiapo	273 (43)	35	1		1
Manuel Renaud	92 (15)	21	0.50 (0.28 - 0.87)		0.67 (0.36 - 1.26)
Padre Barral	186 (30)	20	0.49 (0.32 - 0.76)		0.61(0.37 - 1.01)
Santos de Abalgas	79 (13)	17	0.38 (0.20 - 0.72)		0.52(0.25 - 1.07)
Cooking method				<0.01	
Gas	123 (20)	15	1		1
Gas and woodsmoke	203 (31)	20	1.38 (0.76 - 2.51)		1.44 (0.78 - 2.67)
Woodsmoke	299 (48)	35	3.05 (1.77 - 5.24)		2.12 (1.18 - 3.84)
Gasoline and woodsmoke	3 (1)	0	#		#
Wall status of house				<0.01	
With walls	431 (69)	20	1		1
Without walls	196 (31)	40	2.66 (1.84 - 3.85)		1.83 (1.20 - 2.79)
Unknown	1 (0)	0	#		#
Number of cigarettes per day smoked in household				<0.01	
0	318 (51)	28	1		1
1 - 10	281 (45)	22	0.73 (0.50 - 1.06)		0.89 (0.57 -1.38)
> 10	27 (4)	56	3.30 (1.48 - 7.32)		2.69 (1.11 - 6.48)
Relation of smoker to child				0.79	
Father	206 (33)*	25	1		
Brother	13 (3)	17	0.55 (0.12 - 2.58)		
Grandparents	83 (13)	29	0.58 (0.67 - 2.02)		
Mother	25 (4)	20	0.72 (0.26 - 1.95)		
Others	65 (10)	29	0.77 (0.61 – 2.04)		

* Percentage of the total of 630 children (in some cases more than one smoking relation per child was present).

In univariate analysis, the prevalence of wheezing in the last 12 months was positively associated with the total number of cigarettes that were smoked daily in the household (p < 0.01) and negatively associated with age (p < 0.01). A significantly higher prevalence of asthma symptoms was found in children from households using wood compared with households where gas was used for cooking (p < 0.01). Furthermore, prevalence of asthma symptoms was significantly higher in boys than in girls (p = 0.037) and in children living in houses without walls compared with children living in houses with walls (p < 0.01). There was a significant difference in the prevalence of asthma symptoms (range, 17%-35%) between regions (p < 0.01).

The multivariable analysis (Table 2) was adjusted for all variables associated with a p ≤ 0.25 with asthma symptoms in the univariate analysis, i.e. age, sex, region, cooking method, wall status of the house and the number of cigarettes smoked per day in the household. In multivariable analysis, all variables significantly influencing the prevalence of asthma symptoms univariately remained statistically significant, except for sex and region (Table 2).

## Discussion

We observed a prevalence of asthma symptoms of 26 percent in indigenous Warao children aged two to ten years. This prevalence is within the range of 8.6 - 32 percent of prevalence rates found in the ISAAC study performed in 2000 in Latin America [3]. Lai et al. reported a prevalence of asthma symptoms of 10 to 20 percent in Venezuelan schoolchildren. They did not, however, report whether included children lived in rural or urban areas [4]. The only other study assessing the prevalence of asthma symptoms in rural indigenous Latin American children was performed in the highlands in Guatemala, where a low prevalence of asthma symptoms of 3.3 percent was observed in children aged four to six years [11]. We observed an increased prevalence of asthma symptoms in children living in households using wood fires for cooking compared with children living in households where gas was used for cooking (AOR 2.12, 95% CI 1.18 - 3.84). Other studies addressing the relationship of asthma symptoms and biomass smoke showed similar results. Schei et al. observed a higher prevalence of asthma symptoms in children exposed to smoke from open fires compared to children exposed to cooking with *planchas*, a local type of chimney stove used in rural Guatemala (AOR 3.4, 95% CI 1.3 - 8.5) [11]. Melsom et al. observed a higher prevalence of asthma symptoms in Nepalese children 11 to 17 years of age who were exposed to cooking on open fires compared to children who were exposed to cooking on gas or kerosene stoves (AOR 2.2, 95% CI 1.0 - 4.5) [12]. In a case–control study in Nairobi a higher prevalence of asthma symptoms in children sleeping in bedrooms with damage from dampness compared to children sleeping in bedrooms without damage from dampness was observed (AOR of 4.9, 95% CI 2.0 - 11.7) [13]. However, several other studies did not observe a significant association between asthma symptoms and indoor wood smoke. Noorhassim et al. performed a cross-sectional study in 1007 children aged one to 12 years in Malaysia and did not observe an association of asthma symptoms to wood stove cooking, use of mosquito coil or smoking parents [16]. No significant associations of asthma symptoms to cooking fuel type were observed in Malaysian and South African children, while exposure to environmental tobacco smoke at home was associated with an increased likelihood of asthma symptoms [14,18].

Living in a house without walls was positively associated with asthma symptoms in our survey. Furthermore, a significant association of the variable “house without walls” with the variable “cooking on wood” was observed. We think that this association can be explained by the difference in the degree of acculturation between households. Warao people traditionally live in houses without walls. Over the past 20 years, acculturation has led to more Warao families building houses with walls for protection from the weather or for protection of newborns [28]. Thereby, cooking on gas has become increasingly popular among Warao people as this makes heavy wood gathering unnecessary. Less acculturated families usually cook on wood fire and live in houses without walls while more acculturated Warao more often have a house with walls and gas stoves. There are some studies showing a relationship between the level of acculturation and asthma symptoms, but none were performed in indigenous populations. A study performed in the United Kingdom and Scotland showed a higher prevalence of persistent wheezing in children whose fathers’ social class was low and in those living in high poverty index score areas compared to children whose fathers belonged to a higher social class and children from low poverty index score areas (p < 0.001) [29]. Furthermore, a study performed in New York City showed that asthmatics were five times more likely to live in public housing than non-asthmatics [30].

We observed a higher prevalence of asthma symptoms in the children exposed to more than ten cigarettes per day compared to children not exposed to cigarettes (AOR 2.69, 95% CI 1.11 - 6.48). There is ample evidence of a strong positive association of environmental tobacco smoking to asthma symptoms as stated in the Global Asthma Report [31]. Maternal smoking seems to be particularly influential on the prevalence of asthma symptoms in children [21]. The mother was the primary caregiver in 92 percent of the cases in our population, and only four percent of the children in our study had a smoking mother. In our study, fathers were responsible for the highest exposure to tobacco smoke, namely in 67 percent of the children, while they were the primary caregiver in only one percent of the children. In contrast to the findings in other studies, the prevalence of asthma symptoms in our study was not higher in children exposed to one to ten cigarettes daily compared to non-exposed children. We hypothesize that this is due to the finding that the primary caregivers were smokers in very few families in our study and exposure to tobacco smoking is probably not substantial when the people who smoke are not the caregivers.

There is evidence that environmental exposure to triggers, including particulate air pollution, is associated with a non-specific lung irritation effect rather than with asthma. The results of an experimental study using a mouse model of allergic airway inflammation suggest that woodsmoke exposure can exacerbate rather than cause allergic airway inflammation [32]. As no prospective studies assessing the influence of woodsmoke on the development of asthma have been performed, it is not known whether indoor combustion is associated with the development of asthma or with exacerbation of symptoms among asthmatic individuals [33]. The latter hypothesis would suggest that woodsmoke has a non-specific lung irritation effect rather than an etiologic contribution to the development of asthma. An experimental study performed by Riddervold et al. supports this hypothesis since they did not observe a significant effect of wood smoke exposure on lung function or cytokine levels in non-smoking volunteers without bronchial hyper-responsiveness [34].

Preventive measures should be taken to avoid health damage related to the use of open wood fires. Harris et al. observed a 40–73 percent decrease in clinic visit rates for lower respiratory infections in children 0 to 5 years of age in the year 2006 compared to the year 2002, when chimney stoves were installed (p < 0.01) [35]. The introduction of two types of improved stoves in rural communities in Peru resulted in a 42 - 54 percent reduction of personal exposure to particle matter (PM) in women (p < 0.05) [36]. Albalak et al. measured PM in 30 households in rural Guatemala over a period of 8 months. They also found a significant reduction of PM when open fires were replaced by chimney stoves in a rural setting in Guatemala (p < 0.05) [37].

The indoor levels of PM and carbon monoxide (CO) found in studies performed in rural areas are much higher than WHO recommendations of indoor air quality [38,39]. The content of the smoke produced by biomass containing PM and CO is thought to cause the respiratory problems such as asthma and respiratory infections [40]. Bruce et al. investigated the relationship between the time period that children younger than 18 months of age were present in the kitchen during cooking and CO exposure. During two observations, they observed a significantly higher exposure to CO when the child was in the kitchen twice compared with when the child was in the kitchen only once (p < 0.01) [41]. This means that education about the risks of wood smoke, improved chimney stoves and keeping the children out of the kitchen during cooking could play a role in asthma prevention.

There are a number of limitations to our study, some of which are related to the challenging logistics of conducting an epidemiological study in an area with a poor infrastructure characterized by low literacy and poor access to health care. First, we added clarifications adjusted to local terminology to the ISAAC questionnaire. In order to minimize errors in reporting of asthma symptoms in these populations, questionnaires must use terminology familiar to participants [42]. Although these clarifications as well as the study aims and definitions were discussed with community elders, local health care workers and local translators prior to initiation of the survey, it remains possible that the verbal translation of the questions into the native Warao language was not always accurate. This could have led to under- or over-reporting of symptoms when parents did not speak Spanish.

Second, although we performed a multivariable analysis taking into account possible confounders such as age and sex, unmeasured factors may have caused residual confounding. Overcrowding, high parental education levels, a family history of asthma, shorter duration of breastfeeding and having pets in the home have been positively associated with self-reported asthma in other studies using the ISAAC questionnaire [43-48]. The prevalence of asthma symptoms increased in children that were breastfed for less than 6 months in a population-based prospective cohort study of Sonnenschein-van der Voort et al. [47]. As virtually all Warao children are breastfed until at least 12 months of age, it is not likely that the lack of information on duration of breastfeeding has influenced our study results. The same accounts for parental education, as Warao parents generally have not received any formal education. However, the lack of information on a family history of asthma and the presence of pets in the household may have caused residual confounding. A positive association between parental asthma and asthma symptoms in children has been observed in other studies [43,44,48]. It is, however, questionable whether the answers to questions related to a family history of asthma would have been reliable in our study setting. As Warao women usually have eight or more children, families are large and the constant migration of mainly male adults to and from other communities means that families live scattered across the Delta Amacuro. Due to the poor infrastructure and the lack of telephone or internet access, there is very little contact between family members, if any. The role of pets in the development of asthma symptoms is controversial [49]. Although positive associations between pets in the household and asthma symptoms have been described in single-center studies [43,44], no significant association of current cat or dog exposure to asthma symptoms was observed in children under ten years of age in a third phase ISAAC study including children from 98 countries in all parts of the world [50].

A third limitation to our study is the cross-sectional study design. As we measured the prevalence rather than the incidence of asthma symptoms, it remains unknown whether woodsmoke is a risk factor involved in the etiology of asthma symptoms. Belanger et al. performed a review including studies assessing the association of asthma symptoms with indoor combustion sources and concluded that no studies measuring asthma symptom incidence have been published [33]. Prospective birth cohort studies are needed to determine the role of woodsmoke exposure in asthma etiology and the severity of asthma symptoms over time.

Fourth, we measured the prevalence of asthma symptoms anamnestically without objectively assessing lung capacity, for example by spirometry. However, spirometers are not available in the small health posts in the Delta Amacuro and no personal medical records in which spirometry results or asthma episodes could be recorded are kept. As spirometry values are usually normal between attacks of asthma symptoms, the performance of spirometry in our cross-sectional survey would not have been of additional value to the diagnosis of asthma. Furthermore, the ISAAC questionnaire is a validated and widely used instrument for measuring asthma symptoms in rural settings. Yeh et al. observed a significant advantage of the ISAAC video questionnaire compared with the ISAAC written questionnaire for the accurate diagnosis of asthma symptoms compared to the golden standard spirometry [51]. In our survey it was, however, not feasible to use the ISAAC video questionnaire due to limited power supplies.

Finally, the sample size of our study was small in comparison with the ISAAC studies including more than 50,000 children [1,3,52,53]. The ISAAC study performed in Latin America included 88,813 children from nine different Latin American countries [3]. There are, however, several limitations to the interpretation of the ISAAC Latin American data: a) most of the study sites were concentrated in large, mainly urban areas, b) many of the participating countries have levels of socioeconomic development comparable to industrialized nations and c) sampling of participants was not representative of the different socioeconomic strata within each country. There is a relative absence of epidemiologic studies in rural, poor, indigenous populations in Latin America while ten percent of the Latin American population consists of indigenous people [54]. Our study provides estimates of the prevalence rate of asthma symptoms in children in a rural indigenous population in Latin America as well as insight into the factors associated with asthma symptoms in these children.

## Conclusions

We observed a high prevalence of asthma symptoms in Warao Amerindian children two to ten years of age. We also observed a significantly higher prevalence of asthma symptoms in children living in households using open fires for cooking compared with children living in households using gas. Our findings highlight the role of wood smoke as a potential risk factor for having asthma symptoms in children living in rural settings. There is a need for an integrated approach including education aimed at behavioral changes to improve the lung health status of Warao children in Venezuela.

## Abbreviations

AOR: Adjusted odds ratio; CI: 95% confidence interval; CO: Carbon monoxide; ETS: Environmental Tobacco Smoke; ISAAC: The International Study on Asthma and Allergies in Childhood; OR: Odds ratios; PM: Particle matter.

## Competing interests

The authors declare that they have no competing interests.

## Authors’ contributions

SK participated in the design of the study, the collection of data, the statistical analysis, the interpretation of data and drafting the manuscript. LV participated in the design of the study, the collection of data, the statistical analysis, the interpretation of data, drafting the manuscript and revising it critically. EV participated in the collection of data and helped to draft the manuscript. JG participated in the collection of data and helped to draft the manuscript. LR participated in the collection of data and helped to draft the manuscript. PC participated in the collection of data and helped to draft the manuscript. BN advised on patient recruitment and revised the manuscript critically for important intellectual content. PH participated in the design of the study and revised the manuscript critically for important intellectual content. JW participated in the design of the study, coordinated the field work, and revised the manuscript critically for important intellectual content. All authors read and approved the final manuscript.
